# Antarctic thraustochytrids: Producers of long‐chain omega‐3 polyunsaturated fatty acids

**DOI:** 10.1002/mbo3.950

**Published:** 2019-10-21

**Authors:** Carolina Shene, Paris Paredes, Daniela Vergara, Allison Leyton, Marcelo Garcés, Liset Flores, Mónica Rubilar, Mariela Bustamante, Roberto Armenta

**Affiliations:** ^1^ Department of Chemical Engineering and Center of Food Biotechnology and Bioseparations BIOREN Universidad de La Frontera Temuco Chile; ^2^ Centre of Biotechnology and Bioengineering (CeBiB) Universidad de La Frontera Temuco Chile; ^3^ Master Program in Engineering Sciences with specialization in Biotechnology Universidad de La Frontera Temuco Chile; ^4^ Doctoral Program in Sciences of Natural Resources Universidad de La Frontera Temuco Chile; ^5^ Mara Renewables Corporation Dartmouth NS Canada

**Keywords:** docosahexaenoic acid, eicosapentaenoic acid, lipids, marine microorganisms, polyunsaturated fatty acids, Thraustochytriidae

## Abstract

Thraustochytrids have been isolated from different aquatic systems; however, few studies have reported their occurrence in Antarctica. In this study, 13 strains close to strains belonging to the genera *Oblongichytrium*, *Thraustochytrium,* and *Aurantiochytrium* were isolated from seawater samples collected near the Antarctic Base Professor Julio Escudero (S 62°12′57′ E 58°57′35″). Docosahexaenoic acid (DHA) was found in the total lipids of all the isolates; DHA content of the biomass (dry weight) varied between 3.3 and 33 mg/g under the growth conditions for isolation. Five of the Antarctic thraustochytrids were able to accumulate lipids at levels higher than 20% w/w. Two strains, RT2316‐7 and RT2316‐13, were selected to test the effect of the incubation temperature (at 5°C for 14 days and at 15°C for 5 days). Incubation temperature had little effect on the lipid content and biomass yield; however, its effect on the fatty acid composition was significant (*p* < .05). The low incubation temperature favored the accumulation of eicosapentaenoic acid (EPA), palmitic acid and stearic acid in the total lipids of RT2316‐7. Percentage of EPA, DHA and the omega‐6 fatty acid dihomo‐γ‐linolenic acid of total fatty acids of RT2316‐13 was higher at the low incubation temperature. RT2316‐13 accumulated the highest lipid content (30.0 ± 0.5%) with a carbon to nitrogen mass ratio equal to 16.9. On the contrary, lipid accumulation in RT2316‐7 occurred at high concentration of the nitrogen sources (monosodium glutamate or yeast extract). The capability to accumulate lipids with a fatty acid profile that can be tuned through cultivation temperature make the Antarctic thraustochytrid RT2316‐13 a candidate for the production of lipids with different uses.

## Graphical abstract

Marine thraustochytrids are large‐celled oleaginous microorganisms known for their ability to produce docosahexaenoic (DHA) and eicosapentaenoic (EPA) ω‐3‐fatty acids. In this work, strains close to members of the genera Oblongichytrium, Thraustochytrium, and Aurantiochytrium were isolated from seawater samples collected in the Antarctic. DHA was found in the total lipids of all the isolates; DHA content of the biomass (dry weight) varied between 3.3 and 33 mg/g under the growth conditions for isolation. Temperature had little effect on the lipid content and biomass yield; however, its effect on the fatty acid composition was significant. The incubation at 5°C increased the percentage of EPA and omega‐6 fatty acid dihomo‐γ‐linolenic acid (DGLA) of total fatty acids in the strains RT2316‐7 and RT2316‐13, respectively. These strains are promising candidates for the biotechnological production of lipids.

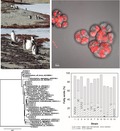

## 1 INTRODUCTION

Docosahexaenoic acid (DHA, C22:6n3) is one of the long‐chain most unsaturated omega‐3 fatty acid, which is normally found in marine oily fish. DHA is also the main omega‐3 fatty acid in the human brain where it is synthesized from the essential fatty acid α‐linolenic acid (ALA; 18:3n3). (Weiser, Butt, & Mohajeri, 2016) However, the rate of conversion of ALA into DHA would not provide the required levels for optimal neurological functions, and its intake is suggested by health organizations.

Marine thraustochytrids are known because of their ability to synthesize DHA which is found distributed in cell membrane lipids and neutral lipids (triacylglycerols). The role of DHA in the cells of thraustochytrids is not fully understood but should contribute to the survival under the prevailing conditions of their habitats. One of those conditions is salinity since most of DHA producer strains requires sea salts, albeit at different levels. Few studies have evaluated the effect of salt concentration on fatty acid desaturation in thraustochytrids. (Burja, Radianingtyas, Windust, & Barrow, 2006; Kang, Jeh, Seo, Chun, & Hur, 2007) Enhanced tolerance to salt stress has been demonstrated in *Saccharomyces cereviciae (*Rodríguez‐Vargas, Sánchez‐García, Martínez‐Rivas, Prieto, & Randez‐Gil, 2007) and *Synechococcus* sp. (Allakhverdiev, Kinoshita, Inaba, Suzuki, & Murata, 2001) genetically engineered to increase the unsaturation of their fatty acids. The activation of the Na^+^/H^+^ antiport system via enhanced fluidity of membranes is a plausible explanation. (Allakhverdiev et al., 2001).

The ability of microorganisms to adjust fluidity of cell membrane is essential to maintain important metabolic functions such as permeability, substrate uptake and the electron transport chain. (Denich, Beaudette, Lee, & Trevors, 2003) Unsaturation of fatty acids is a very effective mechanism for cell adaptation to low temperatures, because it has a higher effect on lipid fluidity. Changes in the unsaturation of fatty acids can be realized by de novo synthesis or desaturation of already existing fatty acids. (Russell, 1984) Low temperature would be a selection pressure for the evolution of microorganisms able to produce PUFA. (Nichols, Nichols, & McMeekin, 1995) In fact, sea ice surrounding Antarctica is one habitat where many PUFA producer bacteria have been isolated. (Nichols, 2003) The analysis of a large dataset of fatty acid profile from marine and terrestrial organisms led to the conclusion that compared to terrestrial organisms eicosapentaeonic acid (EPA, C20:5n3) and DHA are more abundant in marine organisms. (Colombo, Wacker, Parrish, Kainz, & Arts, 2017) In addition, the content of PUFA in polar and temperate marine organisms is higher than in organisms from the tropics.

To date, the main source of EPA and DHA is fish oil. Fatty acid composition of fish oil is very dependent on the fish (Sargent, 1997), and both the season (Bandarra, Batista, Nunes, Empis, & Christie, 1997) and latitude (Colombo et al., 2017) of the capture. Fish oil contains relative high concentrations of marine pollutants, especially persistent organic pollutants. (Sun et al., 2018) Therefore, a refining process is necessary to produce fish oil for human consumption. The biotechnological production of DHA using heterotrophic microorganisms offers various advantages; (a) it is a sustainable process that could be based on cheap and renewable carbon and nitrogen sources, (b) under controlled growth conditions lipid yield and quality can be reproduced, and (c) it is free of pollutants.

Considering the biotechnological potential of thraustochytrids for the production of DHA, the aim of this study was to isolate novel strains from seawater and marine sediment samples collected in the Chilean Antarctic peninsula, to identify the isolates at a molecular level, and to evaluate the biomass and DHA production. The effect of incubation temperature on the biomass and lipid production, and the fatty acid composition of the total lipids was evaluated for two of the Antarctic thraustochytrids.

## 2 MATERIALS AND METHODS

### 2.1 Isolation of thraustochytrid strains

Samples of water and sediments were collected in sterile bottles from the coast near the Antarctic Base Professor Julio Escudero (S 62°12′57″ E 58°57′35″) in December 2016. The samples were supplemented with sterile pine pollen (ca. 15 mg/200 ml) and kept at 5°C. (Gaertner, 1968) After 15 days, pollen grains with attached microorganisms were collected by filtration (0.45 μm) and dispersed on solid medium. The composition of the solid medium was as follows: glucose (Merck) 1 g/L, yeast extract (BBL^™^, Becton, Dickinson and Co.) 6 g/L, and agar (Merck) 15 g/L, in artificial seawater 50% v/v. (Shene et al., 2013) Streptomycin sulfate and penicillin G (0.3 g/L of each) (Sigma) were used to avoid proliferation of bacteria. Incubation of the plates was carried out at 5 and 20°C until the colonies were visible. Individual colonies from plates incubated at 5 and 20°C were subcultured on solid medium at the same temperature until pure isolates were obtained (microscopic inspection). The isolated microorganisms were grown in a liquid medium (glucose 2 g/L, yeast extract 6 g/L, monosodium glutamate (Merck) 0.6 g/L, in ASW 50% v/v) at the temperature at which they were isolated. Stock cultures, prepared by transferring 0.5 ml of the grown culture into a microtube containing sterile glycerol (0.5 ml), were maintained at −80°C.

### 2.2 Microscopic analysis

Cell morphology was observed using an optical microscope (Olympus CX22LED) with 40× magnification. Lipid distribution in cells stained with BODIPY^®^ 581/591 (Thermo Fisher Scientific, Molecular Probes^™^) (300 μl of the cell culture incubated with 10 μl of the fluorophore) was observed using an Olympus Fluoview (FV) 1,000 confocal laser microscopy (Olympus). The images were obtained with a 100× magnification (immersion oil) using the software included in the equipment (FV10‒ASW 2.0).

### 2.3 DNA extraction and identification of isolates

The UltraClean^™^ Kit (Mo Bio Laboratories, Inc.) was used for DNA isolation. DNA was quantified in an Infinity^R^ 200 Pro microplate reader (Tecan). The sequence of the 18S rRNA gene was amplified using the conditions for PCR described by Mo et al. with some modifications. (Mo, Douek, & Rinkevich, 2002) The primers were as follows: forward (F) FA1 (5′‐AAAGATTAAGCCATGCATGT‐3′), and FA2 (5′‐GTCTGGTGCCAGCAGCCGCG‐3′), and reverse (R) RA2 (5′‐CCCGTGTTGAGTCAAATTAAG‐3′) and RA3 (5′‐CAATCGGTAGGTGCGACGGGCGG‐3′). Two PCR was performed to obtain more specific amplification products. PCR program for FA1‐RA2 was 3 min 95°C, 30 cycles of 1 min 94°C, 1 min 61°C, 1 min 72°C, and 10 min 72°C. PCR program for FA2‐RA3 was 3 min 95°C, 30 cycles of 1 min 94°C, 1 min 69°C, 1 min 72°C, and 10 min 72°C. Purification of PCR products was carried out following the instructions of the E.Z.N.A Gel Extraction kit (Omega Bio‐tek Inc.). Sanger sequencing was performed by Macrogen Inc. The results were assembled using the Geneious 4.8.4 program (Biomatters Ltd.) and compared with sequences in EMBL/DDBJ/PDB/GenBank databases by BLASTN 2.2.21 analysis. All the sequences were aligned using the Geneious 4.8.4 software, taking the sequence of *Thraustochytrium striatum* AB022112.1 (NCBI) as reference. A phylogenetic tree was generated by phylogeny.fr (Dereeper et al., 2008) (http://www.phylogeny.fr), using MUSCLE, ProtDist/FastDist + BioNJ (distance‐based method), and TreeDyn for multiple sequence alignment, tree construction, and tree visualization, respectively.

### 2.4 Biomass growth and lipid production

The inoculum to the culture experiments was prepared in Erlenmeyer flasks (250 ml) containing 100 ml of the sterile medium (glucose 20 g/L, yeast extract 6 g/L, monosodium glutamate 0.6 g/L, in 50% v/v ASW) to which 5 ml of the grown culture, described in 2.1, were added. At the inoculation time, 2.4 ml of a trace mineral solution, 360 μl of vitamin I solution, and 360 μl of vitamin II solution, sterilized by filtering under 0.2 μm, were added; composition of trace and vitamin solutions are given elsewhere. (Shene et al., 2013) Incubation was done in an orbital shaker (Zhicheng, 215 g) at the temperature at which the isolates were obtained. All the culture experiments were made in duplicate. The biomass was recovered by centrifugation (7,000 × g, 4°C, 10 min), lyophilized and stored at −20°C until analysis. Results are presented as average ± standard deviation.

The growth curve was obtained for two of the isolated thraustochytrids: RT2316‐7, similar to *Thraustochytriidae* sp. SEK 691 (99% identity), and RT2316‐13 similar to *Oblongichytrium* sp. BAFCult 3,519 (99% identity). The strain RT2316‐7 was selected because of the high DHA percentage of the total fatty acids while strain RT2316‐13 was selected because of its capability to produce abundant biomass with a high lipid content. Erlenmeyer flasks (250 ml) containing 100 ml of the sterile medium described above, were inoculated with 5 ml of a culture grown under the same conditions. Incubation was performed in an orbital shaker (215 g) at 5°C for 14 days, and at 15°C for 5 days. Every 24 hr (15°C incubation) or 48 hr (5°C incubation), two flasks were sacrificed for the analysis of biomass concentration (dry weight), the lipid content of the biomass, and residual glucose concentration. The growth curve experiment for each strain was made in duplicate.

Biomass concentration was gravimetrically measured after a known volume of the grown culture was centrifuged (6,000 × g, 4°C, 10 min), washed with distilled water, and lyophilized. The plot of natural logarithm of the biomass concentration versus time was used to determine the specific growth rate. The residual glucose concentration was measured by HPLC using a Bio‐Rad Aminex HPX‐87H column (300 mm length × 7.8 mm internal diameter), kept at 65°C in an Alliance Waters e2695 Separation Module (Waters Inc.). The mobile phase was sulfuric acid (5 mM) at rate of 0.6 ml/min. Detection was made by refractive index (Waters Inc.). A calibration curve was built with glucose standard solutions.

### 2.5 Effect of the medium composition on the biomass and lipid production

The effect of the initial glucose concentration (0, 10, 20, 30, and 40 g/L), yeast extract concentration (0, 3, 6, and 9 g/L), and monosodium glutamate concentration (0.0, 0.3, 0.6, and 0.9 g/L) on the biomass concentration and lipid content of RT2316‐7 and RT2316‐13 after 5 d incubation at 15°C was determined. The effect of each factor (concentration of glucose, yeast extract, and monosodium glutamate) was evaluated by changing one factor at the time. Experiments were carried out in duplicate as described in 2.4.

### 2.6 Lipid content and fatty acid composition

Lipids in the freeze‐dried biomass (50 mg) were extracted (1 hr, 215 g) with 9.5 ml of a solvent system chloroform/methanol/phosphate buffer (50 mM, pH 7.4) in the volume ratio of 2.5:5.0:2.0. (Bligh & Dyer, 1959) The mixture was transferred to a conical separating funnel containing 2.5 ml of chloroform; after mixing, 2.5 ml of phosphate buffer was added. The lipids in the chloroform layer were gravimetrically measured after the solvent was evaporated at room temperature in a fume hood. The lipids were methylated with alkaline methanol (2 M KOH in methanol) and extracted into petroleum ether; the ratio of alkaline methanol to petroleum ether was 1:10 by volume. Fatty acid methyl esters in the petroleum ether layer were collected after centrifugation (10,000 × g, 4°C, 5 min). The fatty acid composition of the lipids was determined by gas chromatography. (Shene et al., 2019) Fatty acid methyl esters were identified and quantified with a 37‐component standard FAME Mix (Supelco).

### 2.7 Statistical analysis

One‐way ANOVA was used for data analysis. Duncan multiple range test was used for mean comparison at a level of 5%.

## 3 RESULTS

### 3.1 Isolation and identification of Antarctic thraustochytrids

**Figure 1:**
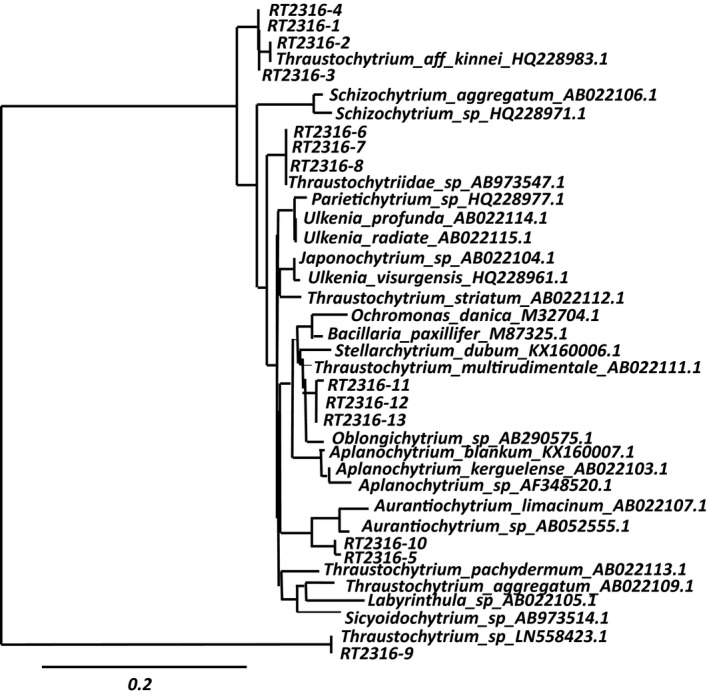
Phylogenetic analysis of the Antarctic thraustochytrids isolated from samples collected from the coast near the Antarctic Base Professor Julio Escudero (S 62 ° 12 '57' 'E 58 ° 57' 35 '') in December 2016 based on data from the nucleotide sequence of 18S rRNA gene. The phylogenetic tree was generated by phylogeny.fr (Dereeper et al., 2008) (http://www.phylogeny.fr), using MUSCLE, ProtDist/FastDist + BioNJ (distance‐based method), and TreeDyn for multiple sequence alignment, tree construction, and tree visualization, respectively

**Figure A1:**
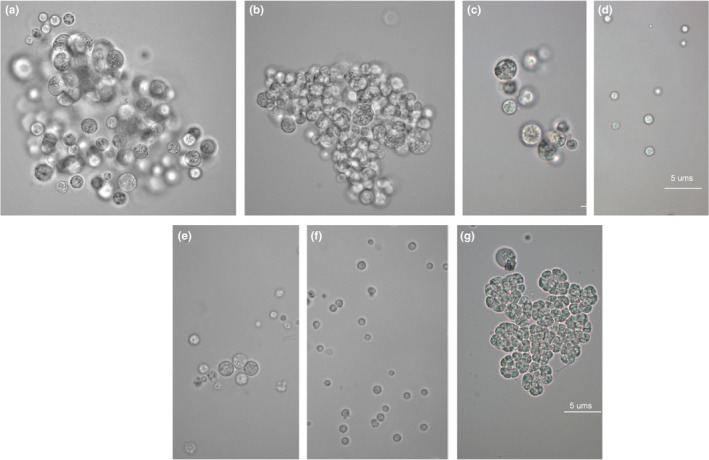
Optical micrograph of some of the Antarctic thraustochytrids isolated from samples collected from the coast near the Antarctic Base Professor Julio Escudero (S 62°12'57' E 58°57'35''). (a) RT2316‐12, (b) RT2316‐11, (c) RT2316‐13, (d) RT2316‐8, (e) RT2316‐6, (f) RT2316‐10, and (g) RT2316‐5

Thirteen thraustochytrid strains were isolated and molecularly identified through 18S rRNA sequencing (Table A1). A phylogenetic tree constructed with the sequences is shown in Figure 1; for comparison purposes, the phylogenetic tree also included the sequence of a *Thraustochytrium striatum* previously isolated. (Shene et al., 2019) The isolates formed four groups of closest relatives and the more distantly related was RT2316‐9. Microscopic observation revealed that some strains grew forming cell aggregates while in other cells were found isolated (Figure A1). Another morphological characteristic that allowed to differentiate the isolates was the presence of zoospores after transferring a grown culture to fresh medium; this was observed in strains RT2316‐5, RT2316‐10, RT2316‐11, RT2316‐12, and RT2316‐13. Under the growth conditions used, strains RT2316‐5, RT2316‐10, RT2316‐7, and RT2316‐8 exhibited an orange pigmentation.

### 3.2 Biomass concentration and lipid content of the biomass of isolated strains

**Figure 2:**
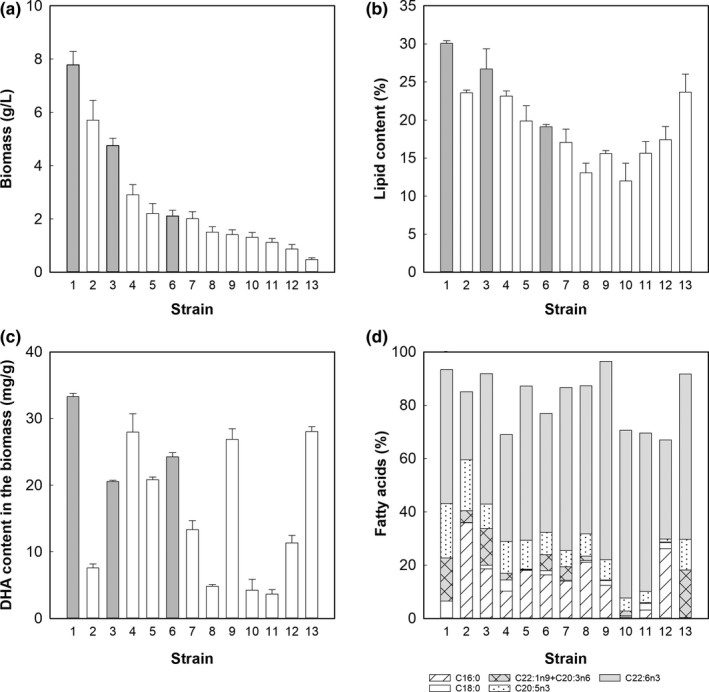
Biomass concentration (a), lipid content of the biomass (b), fatty acid composition of the total lipids (c), and DHA content of the biomass of the Antarctic thraustochytrids (d). Gray and white bars in a, b, and c correspond to the strains cultivated at 5 and 20°C, respectively. The strains are (1) RT2316‐13, (2) RT2316‐12, (3) RT2316‐11, (4) RT2316‐4, (5) RT2316‐6, (6) RT2316‐8, (7) RT2316‐7, (8) RT2316‐9, (9) RT2316‐3, (10) RT2316‐2, (11) RT2316‐1, (12) RT2316‐5, (13) RT2316‐10

To evaluate the production of biomass and lipids, the Antarctic thraustochytrids were cultivated in a glucose–yeast extract–monosodium glutamate medium. The incubation temperature was that at which isolation was made (5 or 20°C). The strains RT2316‐12 and RT2316‐13, incubated at 5°C, reached biomass concentrations higher than 5 g/L (Figure 2a). Among the strains isolated and cultivated at 20°C, RT2316‐11 and RT2316‐4 reached biomass concentrations higher than 2.5 g/L. The lowest biomass concentration (<0.5 g/L) corresponded to RT2316‐10 (Figure 2a).

The lipid content of the biomass of seven isolates was higher than 20% w/w (Figure 2b), and the highest lipid content (30.1 ± 0.4% w/w) was that of RT2316‐13. On the other hand, the lipid content of strains RT2316‐11 (26.7 ± 2.7% w/w, cultivated at 20°C), RT2316‐10 (23.7 ± 2.4% w/w cultivated at 20°C), RT2316‐12 (23.6 ± 0.4% w/w, cultivated at 5°C), and RT2316‐4 (23.1 ± 0.7% w/w cultivated at 20°C) presented no significant differences (*p* > .05).

The content of DHA in the dry biomass varied between 3.3 (in RT2316‐1) and 33 mg/g (in RT2316‐13) (Figure 2c). Fatty acid composition of the total lipids extracted from the biomass of the Antarctic thraustochytrids is shown in Figure 2d. The lipids of all the strains contained EPA and DHA; DHA percentage ranged from 25.5% (in RT2316‐12) to 74.5% (in RT2316‐3) while EPA percentage varied between 1.2% (in RT2316‐5) and 20.5% (in RT2316‐13). The highest percentage of palmitic acid and stearic acid was found in the lipids of RT2316‐2 and RT2316‐1, respectively.

### 3.3 Effect of incubation temperature on biomass, lipids, and DHA production by two selected strains

The strains RT2316‐13 and RT2316‐7 were selected to study the effect of incubation temperature on biomass, lipids, and DHA production. Selection was made based on the high lipid content of RT2316‐13 (Figure 2b) and the high DHA percentage of the total fatty acids of RT2316‐7 (Figure 2d). Strains RT2316‐13 and RT2316‐7 were isolated at different temperatures (5 and 20°C, respectively) and although both were able to grow at 5°C (the low temperature), RT2316‐13 was not able to grow at 20°C. Because of this, the high temperature was set at 15°C.

**Figure 3:**
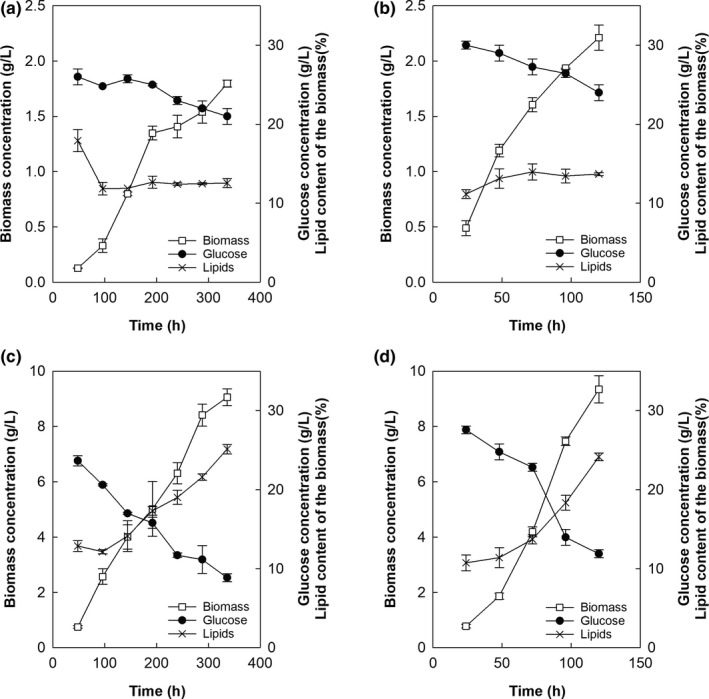
Growth curve (□) of the Antarctic thraustochytrids RT2316‐7 (a and b) and RT2316‐13 (c and d) cultured at 5°C (a and c) and 15°C (b and d). Concentration of glucose (•) and the total lipid content of the biomass (×) are also shown

At the low temperature, the growth curve of RT2316‐7 exhibited two phases (Figure 3a). The specific growth rate decreased from 1.7 × 10^−2^ hr^−1^ (48‒192 hr) to 2.0 × 10^−3^ hr^−1^ (192‒336 hr). Only 25.9% of the initial glucose was consumed during the 14 days incubation, and the final biomass concentration was 1.8 g/L (Figure 3a). The lipid content of the biomass presented a significant reduction (from 17.9 ± 0.4 to 11.9 ± 0.8% w/w) after 96 hr with no significant changes (*p* > .05) after this time. At 5°C, RT2316‐13 grew at a specific growth rate of 5.9 × 10^−3^ hr^−1^ (Figure 3c). Glucose was consumed (64.6%) as the biomass concentration (from 0.7 ± 0.0 to 9.1 ± 0.4 g/L) and lipid content (from 12.9 ± 0.7 to 25.1 ± 0.6% w/w) increased (Figure 3c).

At the high incubation temperature, RT2316‐7 grew at a specific growth rate of 7.8 × 10^−3^ hr^−1^ (between 48 and 120 hr) reaching a final biomass concentration of 2.2 ± 0.1 g/L with a lipid content of 13.7 ± 0.2% w/w (Figure 3b). Under this growth condition, 24.8% of the initial glucose was consumed (Figure 3b). Compared to the low incubation temperature, the high incubation temperature allowed a 1.8‐fold increase of the specific growth rate of RT2316‐13 (3.2 × 10^−2^ hr^−1^) (Figure 3d). However, the final biomass concentration (9.3 ± 0.5 g/L) and the lipid content of the biomass (24.2 ± 0.5% w/w) (Figure 3d) were not significantly different (*p* > .05) from the values obtained with the low incubation temperature (Figure 3c).

**Figure A2:**
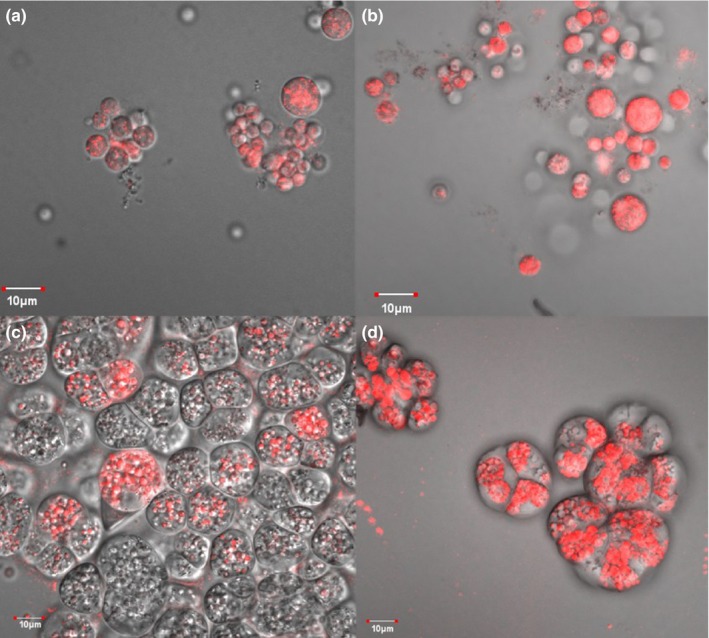
Confocal fluorescence images of the stained cells of the Antarctic thraustochytrids RT2316‐7 and (a and b) and RT2316‐13 (c and d) cultured at 15°C after 24 hr (a and c) and 120 hr (b and d)

Figure A2 compares the distribution of stained lipids in the cells incubated at 15°C harvested after 24 and 120 hr. It was observed that as the incubation time increased, the size of RT2316‐13 cells increased while globular structures corresponding to new cells, accumulated inside. At some point, big cells lysed, releasing the small sized cells. Confocal microscopy images (Figure 2a,b) confirmed that culture conditions allowed the cells of RT2316‐13 to become enriched in lipids during the incubation (Figure 3d). On the other hand, the size of RT2316‐7 cells harvested after 24 and 120 hr presented no important differences (Figure 2c,d). In addition, stained lipids in the RT2316‐7 cells harvested after 24 and 120 hr presented no important differences in agreement with results shown in Figure 3b.

The main fatty acids in the total lipids of RT2316‐13 cultivated at 5°C were dihomo‐γ‐linolenic acid (DGLA, C20:3n6) (19.1 ± 0.1‒56.2 ± 2.2% w/w), EPA (5.4 ± 0.2‒29.8 ± 0.4% w/w), DHA (5.5 ± 0.1–21.4 ± 0.2% w/w), and palmitic acid (9.9 ± 0.2‒13.5 ± 0.2% w/w) (Table 1). The same fatty acids were found in the total lipids of RT2316‐13 cultivated at 15°C. On average, SFA percentage (16.8%–28.5% w/w) of total fatty acids of RT2316‐13 cultivated at 15°C was higher than in the biomass cultivated at the low temperature (13.0%–19.1% w/w). The PUFA/SFA ratio of the total fatty acids of RT2316‐13 cultivated at 5°C was higher than in the total fatty acids of the cells cultivated at 15°C. Incubation time (or culture age) also affected the composition of the total fatty acids of RT2316‐13. As the incubation time increased, EPA and DHA percentage of total fatty acids of cells cultivated at the low and high temperature decreased reaching similar values toward the end of the culture. The incubation temperature had an important effect on the DGLA percentage of the total fatty acids of RT2316‐13. At the low temperature, a significant increase in the DGLA percentage was observed as the incubation time increased. At the high temperature, DGLA was also the main fatty acid (27.6 ± 0.5–38.3 ± 0.5%) but the percentage of linoleic acid (C18:2n6), a precursor of DGLA (omega‐6 PUFA pathway), was higher (on average 2.2‐fold).

**Table 1:** Effect of incubation time (days) and incubation temperature (5 and 15°C) on the fatty acid composition of the total lipids produced by the Antarctic thraustochytrid RT2316‐13. Incubation was made for 14 d at 5°C and for 5 d at 15°C

	**5°C**					**15°C**				
Fatty acid (% w/w)	2 d	6 d	8 d	12 d	14 d	1 d	2 d	3 d	4 d	5 d
Myristic acid, MA (C14:0)	1.6 ± 0.1	2.6 ± 0.0	2.7 ± 0.0	3.1 ± 0.1	3.6 ± 0.0	3.4 ± 0.0	3.5 ± 0.0	4.3 ± 0.0	5.7 ± 0.0	7.6 ± 0.7
Palmitic acid, PA (C16:0)	13.5 ± 0.2	9.7 ± 0.1	7.6 ± 0.0	8.7 ± 1.3	9.9 ± 0.2	11.8 ± 0.6	22.3 ± 0.8	14.5 ± 0.1	13.2 ± 0.2	16.5 ± 1.6
Stearic acid, SA (C18:0)	4.0 ± 0.1	3.0 ± 0.1	2.7 ± 0.0	3.3 ± 0.5	3.3 ± 0.1	1.6 ± 0.1	2.7 ± 0.5	2.0 ± 0.2	2.2 ± 0.1	3.1 ± 0.5
Linoleic acid, LA (C18:2n6)	4.8 ± 0.2	3.8 ± 0.0	5.9 ± 0.0	9.0 ± 1.3	10.9 ± 0.1	14.7 ± 1.0	16.3 ± 2.2	10.0 ± 0.5	15.3 ± 0.3	21.2 ± 1.9
γ‐linolenic acid, GLA (C18:3n6)	2.9 ± 0.0	2.9 ± 0.1	3.8 ± 0.0	4.0 ± 0.0	4.7 ± 0.0	2.9 ± 0.0	3.9 ± 0.3	4.1 ± 0.1	4.2 ± 0.1	4.2 ± 0.0
α‐linolenic acid, ALA(C18:3n3)	0.1 ± 0.0	0.1 ± 0.0	0.1 ± 0.0	0.1 ± 0.0	0.1 ± 0.0	0.5 ± 0.0	0.5 ± 0.1	0.2 ± 0.1	0.3 ± 0.1	0.2 ± 0.1
Eicosadienoic acid, EDA (C20:2)	1.1 ± 0.1	0.9 ± 0.0	1.0 ± 0.0	1.0 ± 0.1	1.2 ± 0.1	1.0 ± 0.1	1.5 ± 0.2	1.5 ± 0.0	1.3 ± 0.0	1.2 ± 0.1
Eicosatrienoic acid, ERA (C20:3n3)	0.3 ± 0.0	0.3 ± 0.0	0.3 ± 0.0	0.3 ± 0.0	0.3 ± 0.0	0.3 ± 0.0	0.6 ± 0.0	0.5 ± 0.1	0.5 ± 0.1	0.4 ± 0.0
Dihomo‐γ‐linolenic acid, DGLA (C20:3n6)	19.1 ± 0.1	45.9 ± 0.2	54.0 ± 0.3	56.2 ± 2.2	53.6 ± 0.4	38.3 ± 0.5	27.6 ± 0.5	31.7 ± 0.1	36.5 ± 0.1	31.0 ± 4.3
Araquidonic acid, ARA (C20:4n6)	0.7 ± 0.0	0.1 ± 0.1	0.2 ± 0.0	0.0 ± 0.0	0.2 ± 0.1	1.8 ± 0.1	0.2 ± 0.1	0.6 ± 0.2	0.2 ± 0.2	0.3 ± 0.0
Eicosapentaenoic acid, EPA (C20:5n3)	29.8 ± 0.4	14.7 ± 0.3	9.8 ± 0.1	6.0 ± 0.8	5.4 ± 0.2	11.4 ± 0.7	10.1 ± 1.9	13.5 ± 0.1	7.4 ± 0.1	4.6 ± 0.5
Docosahexaenoic acid, DHA (C22:6n3)	21.4 ± 0.2	15.2 ± 0.0	11.2 ± 0.5	7.5 ± 0.4	5.5 ± 0.1	11.1 ± 0.5	6.7 ± 1.2	14.1 ± 0.5	10.8 ± 0.3	6.5 ± 1.1
Others	0.8	1.0	0.7	0.9	1.3	1.3	4.1	3.0	2.3	3.1
SFA	19.1	15.3	13.0	15.0	16.8	16.8	28.5	20.8	21.2	27.2
PUFA	80.1	83.7	86.3	84.1	81.9	81.9	67.4	76.1	76.5	69.6
n3‐PUFA	51.6	30.3	21.4	13.8	11.4	23.3	17.9	28.3	19.0	11.7
n6‐PUFA	27.4	52.6	63.9	69.3	69.3	57.6	48.0	46.4	56.1	56.7
PUFA/SFA	4.2	5.5	6.4	5.6	4.9	4.9	2.4	3.7	3.6	2.6

The main fatty acids in the total fatty acids of RT2316‐7 cultivated at 5°C were palmitic acid (13.8 ± 2.5–18.8 ± 1.1% w/w), stearic acid (8.5 ± 0.0–15.8 ± 0.4% w/w), EPA (16.1 ± 1.2–27.2 ± 1.1% w/w), and DHA (25.9 ± 2.0‒39.6 ± 5.0% w/w) (Table 2). The omega‐3 PUFA represented more than 40% of the total fatty acids of RT2316‐7 cultivated at the low temperature whereas the content of omega‐6 PUFA was lower than 14%. At the high incubation temperature, DGLA percentage of total fatty acids was significantly higher (on average 5.7‐fold) than in total fatty acids of RT2316‐7 cultivated at 5°C. DHA percentage of the total fatty acids was not significantly affected (*p* > .05) by the incubation temperature. On the contrary, the high incubation temperature decreased (on average 30%) the EPA percentage of the total fatty acids of RT2316‐7. The SFA percentage of total fatty acids of RT2316‐7 cultivated at 15°C was lower (9.5%–18.1% w/w) than in RT2316‐7 cultivated at 5°C (22.7%–34.9% w/w). On average, total fatty acids of RT2316‐7 cultivated at 15°C had a PUFA/SFA ratio higher than the total fatty acids of RT2316‐7 cultivated at 5°C.

**Table 2:** Effect of the incubation time (days) and incubation temperature (5 and 15°C) on the fatty acid composition of the total lipids produced by the Antarctic thraustochytrid RT2316‐7. Incubation was made for 14 d at 5°C and for 5 d at 15°C

	5°C					15°C				
Fatty acid (%)	2 d	6 d	8 d	12 d	14 d	1 d	2 d	3 d	4 d	5 d
Myristic acid, MA (C14:0)	0.3 ± 0.1	0.4 ± 0.2	0.5 ± 0.0	0.4 ± 0.1	0.2 ± 0.2	0.2 ± 0.0	0.1 ± 0.0	0.0 ± 0.0	0.2 ± 0.1	0.1 ± 0.0
Palmitic acid, PA (C16:0)	18.8 ± 1.0	14.7 ± 0.8	13.8 ± 2.5	14.3 ± 0.0	14.0 ± 0.9	13.4 ± 0.2	15.5 ± 0.4	8.8 ± 0.6	9.0 ± 0.0	8.3 ± 0.3
Stearic acid, SA (C18:0)	15.8 ± 0.4	9.2 ± 0.3	8.9 ± 1.8	10.3 ± 0.5	8.5 ± 0.0	2.1 ± 0.2	2.5 ± 0.6	1.0 ± 0.2	0.7 ± 0.1	1.1 ± 0.5
Linoleic acid, LA (C18:2n6)	5.3 ± 0.1	4.4 ± 0.1	2.4 ± 0.4	1.1 ± 0.2	0.7 ± 0.1	6.0 ± 0.0	2.1 ± 0.0	0.9 ± 0.1	1.0 ± 0.3	1.5 ± 0.1
γ‐linolenic acid, GLA (C18:3n6)	3.4 ± 0.1	0.9 ± 0.1	0.7 ± 0.2	0.3 ± 0.2	0.2 ± 0.1	2.2 ± 0.4	1.1 ± 0.4	1.0 ± 0.3	0.7 ± 0.1	1.1 ± 0.1
α‐linolenic acid, ALA(C18:3n3)	0.5 ± 0.0	0.3 ± 0.1	0.3 ± 0.2	0.1 ± 0.0	0.2 ± 0.1	0.1 ± 0.0	0.2 ± 0.1	0.1 ± 0.0	0.3 ± 0.3	0.0 ± 0.1
Eicosadienoic acid, EDA (C20:2)	0.3 ± 0.0	0.1 ± 0.0	0.1 ± 0.0	0.0 ± 0.0	0.1 ± 0.1	0.3 ± 0.0	0.3 ± 0.1	0.1 ± 0.1	0.2 ± 0.2	0.0 ± 0.0
Eicosatrienoic acid, ERA (C20:3n3)	0.3 ± 0.0	0.1 ± 0.0	0.1 ± 0.0	0.0 ± 0.0	0.0 ± 0.0	0.1 ± 0.1	0.2 ± 0.1	0.0 ± 0.0	0.1 ± 0.0	0.1 ± 0.1
Dihomo‐γ‐linolenic acid, DGLA (C20:3n6)	3.9 ± 1.0	2.8 ± 1.2	4.9 ± 0.3	3.1 ± 2.7	6.2 ± 1.1	25.2 ± 1.4	14.6 ± 0.6	18.0 ± 0.5	29.6 ± 0.1	31.3 ± 1.3
Araquidonic acid, ARA (C20:4n6)	1.2 ± 0.1	0.4 ± 0.2	0.3 ± 0.2	0.6 ± 0.1	0.2 ± 0.2	0.6 ± 0.4	0.4 ± 0.0	0.4 ± 0.4	0.4 ± 0.0	0.3 ± 0.0
Eicosapentaenoic acid, EPA (C20:5n3)	16.1 ± 1.2	21.5 ± 2.2	23.6 ± 0.2	25.4 ± 0.5	27.2 ± 1.1	16.5 ± 1.2	16.2 ± 0.1	15.0 ± 0.4	16.6 ± 0.4	16.4 ± 0.0
Docosahexaenoic acid, DHA (C22:6n3)	25.9 ± 2.0	39.6 ± 5.0	39.4 ± 5.6	39.5 ± 1.7	38.3 ± 1.6	27.9 ± 0.2	39.2 ± 0.5	49.4 ± 0.2	36.9 ± 1.3	36.0 ± 0.8
Others	8.1	5.4	4.9	4.9	4.2	5.5	7.6	5.2	4.4	3.8
SFA	34.9	24.4	23.2	25.0	22.7	15.7	18.1	9.8	9.9	9.5
PUFA	57.0	70.2	71.9	70.1	73.1	78.8	74.2	84.9	85.7	86.7
n3‐PUFA	42.8	61.5	63.4	65.0	65.6	44.5	55.8	64.6	53.8	52.4
n6‐PUFA	13.9	8.6	8.3	5.1	7.4	34.0	18.1	20.3	31.7	34.2
PUFA/SFA	1.6	2.9	3.1	2.8	3.2	5.0	4.1	8.7	8.7	9.1

### 3.4 Effect of medium composition on the production of biomass and lipids

Table A2 shows the effect of glucose concentration, yeast extract concentration, and monosodium glutamate concentration on the biomass concentration and lipid content of RT2316‐7 and RT2316‐13 cultivated at 15°C for 5 d. Biomass concentration of RT2316‐13 was significantly higher (*p* < .05) when the initial glucose concentration was 30 g/L. The increase of glucose concentration from 0 to 30 g/L increased 9.9‐ and 1.6‐fold the biomass concentration and the lipid content of the biomass, respectively. The initial glucose concentration had a small effect on the biomass concentration of RT2316‐7; the highest biomass concentration was obtained with an initial concentration of glucose equal to 20 g/L. The increase of glucose concentration from 0 to 20 g/L increased 1.4‐fold both the biomass concentration and lipid content of RT2316‐7. Yeast extract is a complex mixture of nitrogen compounds (amino nitrogen 6.9% w/w, total nitrogen 11.4% w/w) (BD biosciences, 2015) which also provides minerals and some carbohydrates. The increase of yeast extracts concentration from 0 to 9 g/L allowed increases of 5.7‐ and 8.5‐fold in the biomass concentrations of RT2316‐13 and RT2316‐7, respectively. The lipid content of decreased significantly (*p* < .05) when the initial yeast extract concentration increased; the lowest lipid content of RT2316‐13 (18.4 ± 1.0% w/w) was obtained with the highest concentration of yeast extract (9 g/L). The observed increase in lipid content of RT2316‐7 was not significant (*p* > .05). The effect of monosodium glutamate concentration, at levels tested, was significant (*p* < .05) on the lipid content of both strains; the lipid content of RT2316‐7 increased 1.4‐fold when the monosodium glutamate concentration increased from 0 to 0.9 g/L whereas for RT2316‐13, the highest lipid content of was obtained with a concentration equal to 0.3 g/L.

## 4 DISCUSSION

Thraustochytrids are eukaryotic protists, with abundant occurrence in diverse aquatic ecosystems. It has been reported that thraustochytrids are the most common fungus‐like organisms in the sea, with densities up to 73,000 cells/L in marine sediments. (Raghukumar, 1992) In Antarctic waters, lower densities (5–100 cells/L) have been reported. (Bahnweg & Sparrow, 1974) Riemann and Schaumann reported a dense occurrence of thraustochytrid‐like cells in the lower section of a fast ice core drilled close to the southern shelf ice margin of the Weddell Sea in the Antarctic. (Riemann & Schaumann, 1993) To our knowledge, only three reports have described and characterized strains belonging to the genera *Thraustochytrium* and *Aplanochytrium* in Antarctic samples. (Bahnweg & Sparrow, 1974; Caamaño et al., 2017; Moro, Negrisolo, Callegaro, & Andreoli, 2003) In this work, strains close to members of the genera *Oblongichytrium*, *Thraustochytrium,* and *Aurantiochytrium*, were isolated; in addition, 3 strains close to members of Thraustochytriidae family were isolated. All the isolates were able to produce lipids containing DHA. Nevertheless, significant differences in biomass concentration were observed when these were cultivated in the glucose‒yeast extract‒monosodium glutamate medium not optimized for the production of lipids. Most of the Antarctic thraustochytrids were able to reach biomass concentrations higher than British isolates (ca. 0.78 g/L) and with higher lipid content. (Marchan et al., 2017) Among the Antarctic thraustochytrids, five strains (RT2316‐10, RT2316‐4, RT2316‐11, RT2316‐12, and RT2316‐13) were able to accumulate lipids at levels higher than 20% w/w suggesting that these would be oleaginous strains.

Of the two Antarctic thraustochytrids selected to characterize the lipid production and the lipid composition as a function of incubation time at different temperatures, RT2316‐7 is particularly attractive because of the high DHA percentage of the total fatty acids. Nevertheless, because of the scarce glucose uptake, the design of a medium suitable for biomass and lipid production will be necessary. Compared to RT2316‐7, the strain RT2316‐13 is characterized by a higher glucose affinity, a higher specific growth rate, and a higher content of lipids, which compensate the lower DHA percentage of the total fatty acids. The fatty acid composition of RT2316‐7 and RT2316‐13 might suggest that EPA and DHA are synthesized through a pathway different from that used for the synthesis of other PUFA. In thraustochytrids, long‐chain PUFA can be synthesized via the aerobic desaturase/elongase pathway or the anaerobic polyketide synthase (PKS‐like PUFA synthase). (Metz, 2001) The former comprises a series of alternating desaturation and elongation steps acting on SFA produced in fatty acid synthase pathway. (Matsuda et al., 2012) The PKS‐like PUFA synthase is a multiple enzyme system in which reiterative cycles of condensation, reduction, dehydration, and isomerization steps, introduce the double bounds as the fatty acid chain is elongated. Compared to the aerobic desaturase/elongase pathway, the PKS‐like PUFA synthase pathway would produce relatively pure end‐products.

It was noted that the incubation temperature and time have dramatic effects on the fatty acid composition of RT2316‐13. In a batch culture, the incubation time involves all the changes in the composition of the medium that occur as biomass grows. On the other hand, incubation temperature determines the rate of enzymatic reactions and the specific growth rate. At 15°C, the specific growth rate of RT2316‐13 was 5.4‐fold higher than the specific growth rate at 5°C. At the low temperature, EPA and DHA were the main fatty acids in the total fatty acids of the initially harvested biomass whereas at the high temperature the percentage of omega‐6 PUFAs (linoleic acid and DGLA) was higher. Since the two omega‐6 PUFAs were also found in the total fatty acids of the biomass cultivated at 5°C, it could be assumed that these are precursors of EPA. For this, a Δ5 fatty acid desaturase acting on DGLA would produce arachidonic acid (found at low levels, Table 1) that can be desaturated to EPA by a Δ17 fatty acid desaturase. On the other hand, the composition of the total fatty acids of RT2316‐13 does not allow to relate the synthesis of DHA from EPA due to the absence of the intermediaries in the Delta 4 pathway: the omega‐6 fatty acids docosatetraenoic acid (C22:4n6) and docosapentaenoic acid (C22:5n6), or the omega‐3 docosapentaenoic acid (DPA, C22:5n3). Thus, DHA would be synthesized through the alternative pathway: DHA‐polyketide synthase using acetyl‐CoA. Since acetyl‐CoA is also the substrate for the synthesis of SFA from which DGLA is synthesized, the two pathways compete for the precursor. A low incubation temperature and a high concentration of nutrients (as in the first days of a batch culture) would favor the initial synthesis of DHA in RT2316‐13. Only one report on the PUFA production by strains similar to RT2316‐13 was found. (Kumon et al., 2008) PUFA synthesized by *Oblongichytrium* sp. SEK347, in a 5 d culture at 25°C, were EPA (7.8%), the omega‐3 docosapentaenoic acid (3.1%), and DHA (22.9%). Because of the presence of C22:5n3 instead of C22:5n6, which is different from many other thraustochytrids, the authors suggested that desaturation and elongation of PUFA in *Oblongichytrium* sp. SEK347 preferentially use the omega‐3 fatty acids as substrates. (Kumon et al., 2008) The capability to accumulate lipids with a fatty acid profile that can be tuned through cultivation temperature make of the Antarctic thraustochytrid RT2316‐13 a candidate for the production of lipids with different uses.

The effect of incubation temperature on the production of DGLA has been reported in a recombinant *Saccharomyces cerevisiae* and *Mortierella alpina* 1S‒4. (Kawashima, Akimoto, Higashiyama, Fujikawa, & Shimizu, 2000; Yazawa et al., 2007) In the fungus, the increase of the cultivation temperature from 26 to 28°C resulted in a 7.7% reduction of the DGLA percentage, while in the yeast the DGLA percentage was the highest (2.74% of total fatty acids) at 15°C and was not observed at 30°C. These results might suggest that the effect of temperature on the activity Δ6 desaturase, responsible for linoleic acid (C18:2n6) desaturation into γ‐linolenic acid (C18:3n6) a precursor of DGLA, from different sources is the same. Although the initial objective of this study was to isolate novel thraustochytrids for the production of DHA, DGLA has also attracted the interest because of its medical applications. (Kawashima et al., 2000) DGLA percentage of the total fatty acids of RT2316‐13 is similar to that reported in a mutant *M. alpina* (39.8 ‒ 43.1% w/w) in which the low Δ5 activity decreases the DGLA desaturation to arachidonic acid. (Kawashima et al., 2000).

The strain RT2316‐7 accumulates lipids with a high content of EPA and DHA that together represent between 44.5% and 64.6% of the total fatty acids. The DHA percentage (27.9‒49.4%) of the total fatty acids of RT2316‐7 is two‐ to fourfold higher than of sardine oil (11%). (Morais et al., 2001) Therefore, this Antarctic thraustochytrid is a promising candidate for the biotechnological production of lipids rich in long‐chain omega‐3 PUFA. The composition of the total fatty acids of RT2316‐7 suggests that DHA is not a product of elongation and desaturation activities on SFA. Incubation temperature (5 or 15°C) has no effect on the DHA percentage of the total fatty acids of RT2316‐7. However, the high temperature promotes the elongation and desaturation of palmitic acid into DGLA.

The capability of microorganisms to grow at low temperatures is attributed to the ability to desaturate fatty acids because the presence of PUFA is relevant to maintain the correct phase of membrane lipids. In agreement with this, the ratio PUFA/SFA of the total fatty acids of RT2316‐13 was, on average, higher in cells cultivated at 5°C than in cells cultivated at 15°C. This pattern would not be exhibited by all microorganisms as it is the case of RT2316‐7. A possible explanation would be that not all PUFA are needed to maintain membrane fluidity at a low incubation temperature. On the other hand, the different lipid content of both strains (ca. 10% in RT2316‐7 and 30% in RT2316‐13) suggests that the extracted lipids from RT2316‐7 correspond to membrane lipids whereas those extracted from RT2316‐13 are both storage and membrane lipids.

Although optimizing culture conditions for the production of DHA was not an objective of the present study, the effect of the concentration of the 3 components of the growth medium on the biomass and lipid production by the two selected strains was analyzed. A high carbon to nitrogen mass ratio is usually effective for increasing the lipid content of oleaginous microorganisms. (Converti, Casazza, Ortiz, Perego, & Borghi, 2009) This effect was observed in RT2316‐13 which accumulated the highest lipid content (30.0 ± 0.5%) with a carbon to nitrogen mass ratio equal to 16.9. On the contrary, lipid accumulation in RT2316‐7 occurred at a high concentration of monosodium glutamate or yeast extract. This is not surprising if it is considered that because of the low glucose uptake an alternative carbon source is consumed by this strain. Some amino acids can be consumed to be converted into pyruvate or intermediates of the citric acid cycle (glucogenic amino acids) while others can be channeled into acetyl‐CoA (ketogenic amino acids), the building block for the synthesis of fatty acids.

Thraustochytrids, similar to strains belonging to different genera able to produce lipids containing DHA, were isolated from water and sediment samples collected at the coast near the Antarctic Base Professor Julio Escudero (S 62°12′57″E 58°57′35″). Interestingly, one of the strains for which the isolation methodology was carried out at 5°C was able to produce the highest biomass concentration with a high lipid content. A low incubation temperature (5°C) increased the percentage of EPA and DGLA of the total fatty acids of RT2316‐7 and RT2316‐13, respectively. A more detailed analysis of the fatty acid composition of membrane lipids (phospholipids, glycolipids, and phosphoglycerides) could show if these changes are the responses of an adaptation mechanism that allow these two strains to grow at low temperatures.

## CONFLICT OF INTERESTS

None declared.

## AUTHORS CONTRIBUTIONS

C. S., M. B., M. R., and M. G. conceived the research project leading to this publication. P. P., D. V., M. G., L. F., and A. L. performed the experiments and prepared the initial draft. C. S., M. R., M. B., and R. A. wrote the manuscript.

## ETHICAL APPROVAL

None required.

## Data Availability

All data are provided in full in the results section and the Appendix of this paper apart from the partial sequences of the small subunit ribosomal RNA gene which are available at the GenBank (https://www.ncbi.nlm.nih.gov/) under the assigned accession numbers given in Table A1.

## APPENDIX A

**Table A1:** Accession number of nucleotide sequences of 18S rRNA gene used for molecular identification of the Antarctic thraustochytrids isolated from seawater and sediment samples collected from the coast near the Antarctic Base Professor Julio Escudero (S 62°12'57' E 58°57'35''), sequence length, percentage of identification, and the closest relative on GenBank

Name	Isolate	Sequence length	Identity (%)	Closest relative on GenBank	Accession^b^
RT2316−1	RT2316−1	1659	99	*Thraustochytrium kinnei* SEK680	MN435148
RT2316−2	RT2316−2	1623	95	*Thraustochytrium aff kinnei* BAFCcult 3,509	MN435154
RT2316−3	T6−22	1,310	91	*Thraustochytrium aff kinnei* BAFCcult 3,489	MH236173
RT2316−4	T6−21	1109^a^	95	*Thraustochytrium aff kinnei* SEK618	MH234496
RT2316−5	20–1–2	1567	96	*Aurantochytrium* sp. BAFCcult 3,545	MH277410
RT2316−6	PA−8‐F	576^a^	98	*Thraustochytriidae* sp. SEK 690	MH234222
RT2316−7	4–1	1041^a^	99	*Thraustochytriidae* sp. SEK 691	MH234382
RT2316−8	5–4	1,293	96	*Thraustochytriidae* sp. SEK 692	MH236175
RT2316−9	RT2316−9	1089^a^	98	*Thraustochytrium* sp. 34–2	MN454229
RT2316−10	21–2	1615	95	*Aurantiochytrium* sp. JC3	MH277411
RT2316−11	18–2	1579	99	*Oblongichytrium* sp. SEK 599	MG711616
RT2316−12	31–2	1,498	99	*Oblongichytrium* sp. SEK 715	MG711605
RT2316−13	1F	1517	99	*Oblongichytrium* sp. BAFCcult 3,519	MG711606

a Indicates that the assembly is less than 1,200 bp.

b https://www.ncbi.nlm.nih.gov/

**Table A2:** Effect of glucose (Gluc), yeast extract (YE), monosodium glutamate (MSG) concentrations, and carbon to nitrogen mass ratio (C: N) on biomass concentration and lipid content of the biomass in 5 d cultures of the Antarctic thraustochytrids RT2316‐13, and RT2316‐7 grown at 15°C

Strain	Gluc (g/L)	YE (g/L)	MSG (g/L)	C: *N* * (g/g)	Biomass (g/L)	Lipids (% w/w)
RT2316−13	0			0.0	0.9 ± 0.2^c^	17.2 ± 1.5^c^
	10			5.5	3.4 ± 0.5^bc^	18.9 ± 0.2^c^
	20	6	0.6	10.9	6.1 ± 0.3^b^	27.5 ± 1.8^ab^
	30			16.4	8.9 ± 0.1^a^	28.2 ± 0.7^ab^
	40			21.8	6.9 ± 0.5^b^	23.8 ± 1.2^b^
	30	0	0.6	241.6	2.0 ± 0.1^c^	27.3 ± 1.5^ab^
		3		30.6	6.6 ± 0.5^b^	26.4 ± 1.5^b^
		6		16.4	8.9 ± 0.1^ab^	28.2 ± 0.7^ab^
		9		11.2	11.3 ± 0.9^a^	18.4 ± 1.0^c^
	30	6	0.0	17.5	11.0 ± 0.1^a^	28.3 ± 1.6^ab^
			0.3	16.9	10.9 ± 0.2^a^	30.0 ± 0.5^a^
			0.6	16.4	11.4 ± 0.1^a^	28.5 ± 1.4^ab^
			0.9	15.8	11.6 ± 0.2^a^	28.5 ± 1.5^ab^
RT2316−7	0	6	0.6	0.0	1.6 ± 0.2^d^	8.6 ± 0.3^c^
	10			5.5	1.9 ± 0.2^cd^	11.9 ± 1.0^a^
	20			10.9	2.2 ± 0.3^c^	11.8 ± 0.5^a^
	30			16.4	2.1 ± 0.2^c^	11.5 ± 0.2^a^
	40			21.8	2.0 ± 0.1^cd^	9.7 ± 0.1^b^
	20	0	0.6	161.0	0.4 ± 0.0^d^	10.4 ± 1.2^b^
		3		20.4	1.7 ± 0.0^c^	11.3 ± 0.9^ab^
		6		10.9	2.2 ± 0.3^b^	11.8 ± 0.5^ab^
		9		7.4	3.4 ± 0.2^a^	12.4 ± 0.9^a^
	20	6	0.0	7.8	2.7 ± 0.2^a^	11.5 ± 0.5^b^
			0.3	7.6	2.7 ± 0.2^a^	11.8 ± 0.3^b^
			0.6	7.4	2.2 ± 0.3^b^	11.2 ± 1.7^b^
			0.9	7.3	2.4 ± 0.2^ab^	15.9 ± 0.4^a^

Note

Different letters in the column of a block indicate significant differences (*p* < .05).

* C: N was calculated as (Gluc × 0.4)/(YE × 0.114 + MSG×14/169.1); nitrogen content of YE 11.4% (BD biosciences, 2015), molecular weight of MSG 169.1 g/mol.
